# Nanomedicine and nanoparticle-based delivery systems in plastic and reconstructive surgery

**DOI:** 10.1186/s40902-023-00383-9

**Published:** 2023-03-30

**Authors:** Jea Giezl N. Solidum, Jeremy A. Ceriales, Erika P. Ong, Eric David B. Ornos, Ruth Joy L. Relador, Elgin Paul B. Quebral, Jose Florencio F. Lapeña, Ourlad Alzeus G. Tantengco, Ka Yiu Lee

**Affiliations:** 1 MD-PhD (Molecular Medicine) Program, College of Medicine, University of the Philippines Manila, Ermita, Manila, 1000 Philippines; 2 College of Medicine, University of the Philippines Manila, Ermita, Manila, 1000 Philippines; 3 Department of Otolaryngology - Head and Neck Surgery, Section of Craniomaxillofacial Plastic and Restorative Surgery, College of Medicine - Philippine General Hospital, University of the Philippines Manila, Ermita, Manila, 1000 Philippines; 4 Department of Physiology, College of Medicine, University of the Philippines Manila, Ermita, Manila, 1000 Philippines; 5 Department of Biology, College of Science, De La Salle University, Manila, 1004 Philippines; 6 Swedish Winter Sports Research Centre, Department of Health Sciences, Mid Sweden University, Östersund, Sweden

**Keywords:** Nanotechnology, Regenerative medicine, Plastic surgery, Reconstructive surgery, Nanomedicine, Delivery systems

## Abstract

**Background:**

Nanotechnology and nanomedicine are rising novel fields in plastic and reconstructive surgery (PRS). The use of nanomaterials often goes with regenerative medicine. Due to their nanoscale, these materials stimulate repair at the cellular and molecular levels. Nanomaterials may be placed as components of nanocomposite polymers allowing enhancement of overall biochemical and biomechanical properties with improved scaffold properties, cellular attachment, and tissue regeneration. They may also be formulated as nanoparticle-based delivery systems for controlled release of signal factors or antimicrobials, for example. However, more studies on nanoparticle-based delivery systems still need to be done in this field. Nanomaterials are also used as frameworks for nerves, tendons, and other soft tissues.

**Main body:**

In this mini-review, we focus on nanoparticle-based delivery systems and nanoparticles targeting cells for response and regeneration in PRS. Specifically, we investigate their roles in various tissue regeneration, skin and wound healing, and infection control. Cell surface-targeted, controlled-release, and inorganic nanoparticle formulations with inherent biological properties have enabled enhanced wound healing, tumor visualization/imaging, tissue viability, and decreased infection, and graft/transplantation rejection through immunosuppression.

**Conclusions:**

Nanomedicine is also now being applied with electronics, theranostics, and advanced bioengineering technologies. Overall, it is a promising field that can improve patient clinical outcomes in PRS.

## Background

Plastics and reconstructive surgery (PRS) is a branch of surgery that aims to recreate both form and function of resected or damaged tissue while maintaining or refining aesthetic appearance [1]. As one of the medical subspecialties with the wealthiest and most vibrant innovative research activity, it has evolved from mainly employing autologous solutions (like skin grafts and local flaps) to allotransplantation and an increasing range of engineered synthetic products [1, 2]. One of the most recent innovations already regularly used in this field is reconstructive microsurgery, which employs microscopes and precise instruments to be able to anastomose until the level of the intricate blood vessels and nerves less than a few millimeters in diameter when doing free tissue transfers, reattachment of severed parts, or composite tissue transfers [3].

Despite such progress, issues remain that can be addressed, and opportunities abound to optimize outcomes further. For example, resourcing donor tissues remains challenging in allotransplantation, and there is always the threat of transplant rejection and immunosuppressive toxicity [2]. Tissue engineering attempts to bypass these limitations, but these technologies have been limited by scalability, integration, and vascularity [2]. Synthetic implants, particularly breast implants, have issues of rupture and leakage. As in other types of surgical procedures, there is also always the risk of infection in PRS, and efforts are continuously being made to decrease infection rates. Finally, even such improvements in surgical technique as in microsurgery mainly focus on the mechanical repair of tissue and neglect the potential benefit from stimulation of the underlying tissue biology to improve healing [1].

Nanotechnology and nanomedicine are promising areas of study that can potentially offer solutions to these unmet clinical needs and provide novel applications in PRS. Nanotechnology involves the understanding, controlling, and using of matter at dimensions roughly 1–100 nm, where unique properties have been elucidated [4]. Meanwhile, nanomedicine refers to the application of nanotechnology materials in diagnosing and treating disease [2]. Nanomedicine is at the intersection of the traditional disciplines of chemistry, biology, medicine, engineering, and material science [4]. It emerged due to a desire to understand biological systems (such as tissue structure, cell membrane proteins, soluble plasma proteins, etc.), which are assembled at the nanoscale, and with the development of the atomic force microscope (AFM) and the scanning tunneling microscope (STM) in 1981 [5], that enabled perceiving biological systems at the atomic level [4].

Given these potential applications, it is unsurprising that the fields of nanomedicine and nanoparticle-based delivery systems have seen an exponential rise in the scale of research generated [5]. Therefore, this paper seeks to provide an overview of the recent developments in nanoparticle-based delivery systems and nanoparticles targeting cells for response and regeneration, specifically in their various roles in tissue regeneration, skin and wound healing, and infection control, particularly in the field of PRS. We also discuss their possible future directions in the field.

## Main text

### Use of nanoparticles in medicine

Nanomedicine has shown promising results in many medical fields like oncology, infectious disease, neurology, and cardiology [6–9]. Cancer diagnostics and therapeutics illustrate the extent of the biomedical application of this emerging technology. Nanomaterials improve biomarker level measurements for early cancer [7]. Due to their high surface area-volume ratio, nanoparticles can be densely covered with detecting agents (e.g., antibodies, aptamer, and targeting peptides) to improve the capture and detection of cancer biomarkers. For instance, magnetic nanoparticles are coated with antibodies specific to epithelial cellular adhesion molecule (EpCAM) for detecting colon, liver, lung, or breast cancer. The enhanced efficiency, specificity, and safety of nanoparticles resulted in their increased use as drug delivery or imaging contrast agents for cancer diagnosis and therapeutics [10].

To combat infectious diseases, the FDA recently approved two liposome-packaged antibiotics: liposomal Amphoterecin B (AmBisome®) and Amikacin liposome inhalation suspension (Arikayce®). Ambisome® is a unilamellar liposome preparation of amphotericin B for fungal infections. The liposomal preparation causes increased peak blood concentration and decreased kidney concentration, thus increasing its efficacy while reducing its toxicity. Arikayce®, another liposomal packaged antibiotic, is an inhalation suspension preparation of amikacin for the nontuberculous bacterium. This preparation allows direct delivery to the lungs for infection clearance and increases intracellular and extracellular bacterial killing. Both preparations exhibit increased efficacy with decreased toxicity [11]. Nanomaterials themselves have antibacterial properties that can be utilized to fight against microbes [12]. For example, copper and silver-zinc oxide nanoparticles generate reactive oxygen species leading to bactericidal effects such as damage to cell membranes, protein dysfunction, and leakage of cellular contents [12]. Vaccine manufacturing can also be improved with nanotechnology. Nanomaterials facilitate the engagement of vital immunological pathways for protective immune responses [8]. Several nanocarriers, including gold, carbon, polymers, and liposomal nanoparticles, facilitate the engagement of crucial immunological pathways to achieve a desired immune response for vaccination against several diseases (e.g., tuberculosis, malaria, human immunodeficiency virus, and rotavirus) [8, 13].

Nanotechnology also has promising utilizations in neurological disorders [6]. Several researchers explored nanoparticle drug delivery systems for enhanced transfer and retention of drugs across the blood–brain barrier (BBB), liposomal, polymeric, and platinum nanoparticles, for instance [14]. Likewise, nanomaterial packaging of agents has been applied for better penetration of brain chemotherapeutic drugs [15]. Diagnosis and treatment of cardiovascular disease can also be improved with nanotechnology. For cardiovascular diseases, preclinical studies demonstrated that using gold nanoparticles could enhance contrast and targeting of atherosclerothrombotic plaques, thrombus, and myocardial scars [16]. Nanoparticles such as liposomes, dendrimers, and micelles are fabricated as nano drug carriers to improve drug half-life and enhance targeting [9]. Self-assembling nano peptides, hyaluronic acid, and alginate hydrogels have been evaluated to act as nanogels of protein or cell therapy for cardiovascular diseases [17]. Nano-coated stents for atherosclerotic materials are studied for their potential to improve biocompatibility [18].

**Fig. 1:**
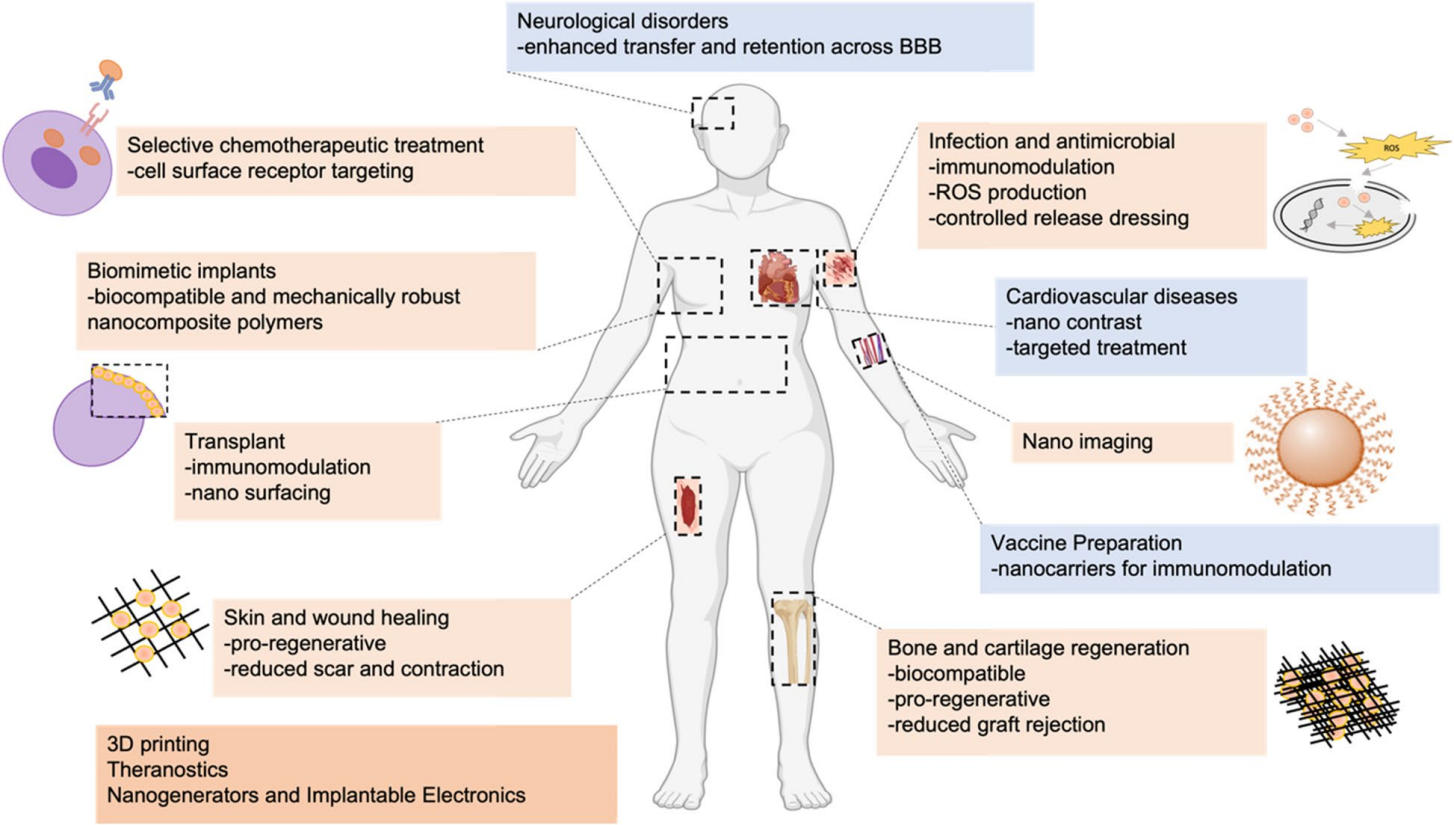
Schematic of nanobased drug delivery applications in facial plastics and reconstructive surgery (PRS), and other fields of medicine. PRS applications are in light orange, while applications to other fields of medicine are in light blue. Figure partially created in Microsoft Powerpoint and BioRender (https://biorender.com/)

**Fig. 2:**
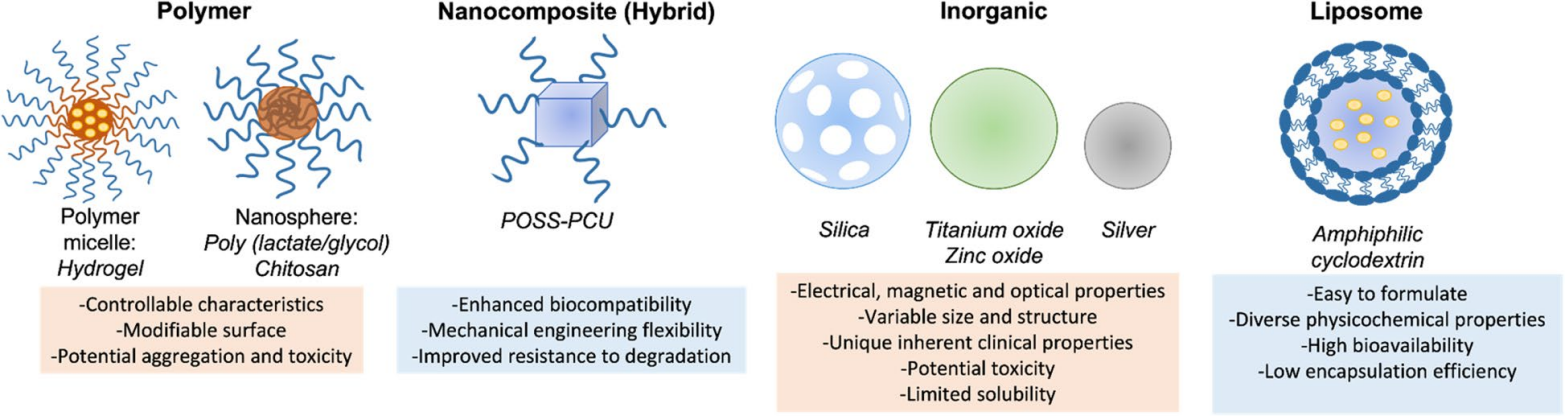
Different classes of nanoparticles used in PRS. Figure created in Microsoft Powerpoint

In the field of PRS, nanomedicine has enabled better visualization and enhancement of biological processes towards enhanced wound healing, tumor identification, and maintenance of tissue viability using different classes of nanoparticles (Figs. 1 and 2), all of which are cornerstones of PRS [2]. In cancer PRS, nanoparticles conjugated with monoclonal antibodies, peptides, or small molecules with high specificity and affinity to target malignant tumor cells are delivered and used to delineate healthy from abnormal cells [19]. Improved implants can be made using biocompatible and mechanically robust nanocomposite polymers. In wound healing and tissue regeneration, nanoparticles can stimulate the body’s repair at a cellular level due to their size and composition [1]. Nanometer surface structures can stimulate accelerated cellular responses and repair by emulating the dimension, geometry, and arrangement of components of natural tissue [4]. Thus, besides nanoparticle-based drug delivery, nanomedicine also capitalizes on the body’s intrinsic healing mechanisms by triggering the physiologic repair response of tissue specific resident tissue cells. This shifts the focus of regeneration in grafting to what is occurring at the cellular and molecular level, as opposed to only the larger spatial defect [1]. In infection control, controlled-release wound dressings for the treatment of wounds have been designed through the electrospinning of nanomaterials [20, 21]. In transplant science, nanoparticle coatings with similar structures or biochemical properties as the host cells/tissues, or the same properties as implantable immunosuppressant delivery, can reduce graft rejection [22, 23].

Translating nanotechnology products for medical use is regulated by the Food and Drug Authority (FDA) [24]. The preclinical efficacy, toxicity, and safety profile of the drug and its physiochemical properties are compiled into an Investigational New Drug (IND) for application to the FDA. Upon approval as an IND, a human trial can ensue. As with other drug products, the human trial has several phases: Phase 1 (dosing toxicity, excretion in health subjects), Phase II (safety, efficacy of a larger group of patients with illness), and Phase III (multi-center, randomized, placebo-controlled). After successful Phase III trials, a New Drug Application (NDA) can then be filed with the FDA for marketing. Phase IV, or post-marketing trials, can also be performed as deemed necessary by the FDA and other clinicians. The process from conception to human use might take 10–15 years and may cost 1 billion USD for the new drug [24]. Translation of nanotechnology products into medical practice incurs some challenges. Safety and medical issues should be investigated appropriately using preclinical toxicology and patient safety trials before they are ultimately approved for commercial use.

### The nanoparticle-based delivery system

#### Implants and cancer

Nanoparticles can make highly selective chemotherapeutic treatment possible by targeting tumors based on their cell surface receptors [21]. In a study by Chen et al. [25], anisamide-targeted nanoparticles were used to systemically deliver c-Myc siRNA into the cytoplasm of B16F10 murine melanoma cells. This nanoparticle formulation targets the sigma receptors found in a melanoma cell. The nano therapy effectively showed suppression of c-Myc expression, partial inhibition of tumor growth, and sensitization of the melanoma cells to paclitaxel resulting in complete inhibition of tumor growth. Additionally, it showed significant inhibition of the growth of the MDA-MB-435 tumor, a metastatic human breast cancer model [25]. In a recent study by Evans et al. [26], the authors formulated and evaluated the therapeutic gene silencing in a three-dimensional (3D) prostate cancer bone metastasis model using anisamide-targeted amphiphilic cyclodextrin nanoparticles by similarly targeting the overexpressed sigma receptor on the surface of prostate cancer cells. Results showed that the targeted nanoparticles showed significantly higher levels of Polo-Like Kinase 1 (PLK1) mRNA knockdown compared to the non-targeted control nanoparticles [26]. PLK1 is a cell cycle key regulator known to be overexpressed in prostate cancer cells and correlated to poor patient outcomes [27].

Selective tumor cell targeting using nanotechnology is best applied in PRS as an imaging technique during the surgical excision of tumors. Nanoparticles conjugated with monoclonal antibodies, peptides, or small molecules with high specificity and affinity to target malignant tumor cells are delivered and used to visualize malignant cells in real time, thereby delineating healthy from abnormal cells resulting in decreased damage to healthy tissues [19]. The use of nanotechnology in cancer detection and diagnosis has been immensely studied, particularly in ex vivo (biological samples) and animal models [7]. Clinical trials are ongoing using different approaches to nanotechnology-based cancer diagnosis [7]. Such an example is a study by Phillips et al. [28] where they used tumor-specific ultrasmall inorganic hybrid nanoparticles (124I-cRGDY-PEG-dots) for the positron emission tomography (PET) scan imaging of metastatic melanoma and malignant brain tumors. Findings showed that the hybrid nanoparticles are safe and well-tolerated in humans [28]. Another ongoing clinical trial is a study by Zanoni et al. [29], where ultrasmall core–shell fluorescent silica (cRGDY-PEG-Cy5.5) nanoparticles were used for real-time image-guided biopsy of sentinel lymph nodes (SLN) in head and neck melanoma. Phase 2 results found this safe for humans. This approach enabled high-sensitivity visualization of SLN, lymph node mapping, deep tissue imaging, including anatomic sites that are difficult to access, and even detecting cancer through intact skin. There was a high concordance rate (90%) compared to the standard-of-care technetium Tc 99 m sulfur colloid. With the intraoperative identification of SLN involvement, extensive and unnecessary dissection of adjacent normal tissues and nerves was prevented [29].

In PRS, biocompatible and mechanically robust nanocomposite polymers have been undergoing active research due to rupture and leakage of currently used implants, such as breast implants [5]. Nanocomposite polymers are just like the typical composite materials comprising two main elements, a matrix, and a filler material for reinforcement, but one or both are of nanoscale size [5]. Tan et al. [30] have developed POSS-PCU (polyhedral oligomeric silsesquioxane poly(carbonate-urea) urethane), a novel nanocomposite material with superior mechanical engineering properties, improved biocompatibility, and enhanced resistance to degradation with various medical applications. POSS-PCU is also valuable for plastic surgery, particularly breast implants. POSS-PCU has already been used in humans as a bypass graft, lacrimal duct, and the first synthetic trachea [30], proving its biocompatibility and mechanical strength. Additionally, Ainslie et al. [31] showed that nanostructured polytetrafluoroethylene (nPTFE) exhibited low macrophage adhesion and inflammatory markers in vitro, possibly leading to its non-immunogenic properties in vivo. With further studies and clinical translation, this finding holds the possibility of decreasing transplant or graft rejection in the future.

#### Bone regeneration

Another promising application for nanomedicine is the facilitation of skeletal regeneration. Bone has an intelligent way of remodeling and repairing itself depending on the changing demands of the body and exposure to stress, injury, and degeneration. The balance of activities of the osteoblasts, osteocytes, and osteoclasts within an extracellular matrix (ECM) allows for self-repair [32]. However, the bone can only fix itself up to a certain limit. Beyond this limit, external help is needed to ensure the proper return of form and function [33]. Conventional surgical interventions, such as internal fixation, autografting, and metal and nonmetal implant application, pose different risks and disadvantages, including but not limited to infection, morbidity, implant rejection, and implant corrosion. Due to the increasing knowledge of the molecular pathways and processes of bone healing, nanomedicine and nanoparticle-based interventions allow for a potentially more specific, targeted, and localized treatment, closely resembling the natural skeletal structures and causing fewer side effects [23].

Qiao and colleagues [23] report the available and currently investigated nanoparticle-based delivery systems for bone regeneration and repair. Nanomaterials can be specifically designed to provide the highest possible biocompatibility to prevent rejection, rapid degradation, and infection while promoting normal physiological repair processes and osseointegration. They can be made of a single component or a combination of different materials (composite) to deliver a synergistic effect and reduce the toxic effect from each component when used solely. Materials can be inorganic, organic, ceramic, or polymeric, depending on the purpose and target site with which the nanoparticles will be used. Nanomaterials can be used as scaffolds, surface-modifying agents, controlled-release drugs, or growth factor delivery systems [23].

Nanofibers are ideal as scaffolds since they provide adequate porosity, surface-to-volume ratio, and space like the natural ECM of the bone that encourages normal cell activity, such as adhesion, proliferation, and differentiation [34]. An example is the hybrid scaffold developed by Hwang et al. in 2016 using poly (Ɛ caprolactone) and gelatin, which exhibited a significant increase in cell infiltration and proliferation, that can potentially improve bone regeneration as well. The surface modification involves the delivery of nanoparticles that can alter the surface of the bone or the graft to enhance osseointegration, prevent graft rejection, or promote cell adhesion to encourage natural bone regeneration [35]. Titanium (Ti) alloy grafts can be coated with nanoparticles to increase resistance to corrosion like the thermal spray developed by Sarao et al. [36], which deposits hydroxyapatite powder with TiO_2_ coating. Some nanoparticles, such as fibroblast growth factor-2 (FGF-2) loaded poly (lactide-co-glycolide) nanoparticles designed by Shim et al. [37], can provide controlled release of growth factors that mimic the expected physiological timing encouraging bone regeneration on the bone-graft interface. EVs can also be used as nanoparticle delivery systems to evade the immune response and ensure targeted delivery as they originated from the cell membrane [23].

#### Cartilage regeneration

Unlike bone, cartilage has no blood supply and local progenitor cell source, which gives it less capacity to repair itself. Hence, interventions involve chondrocyte implantation with mosaicplasty for small lesions, or autologous chondrocyte implantation (ACI) and matrix-induced autologous chondrocyte implantation (MACI) for larger lesions. The idea of nanomaterial engineering and delivery is similar to that of the bone. However, in the cartilage, it may involve the administration of chondrocytes or mesenchymal cells that can be induced with specific growth factors to promote chondrogenesis and cartilage repair. The dual growth factor-loaded chitosan nanoparticle/alginate hydrogel system by Lim et al. [38] provides an excellent example of a complex growth factor delivery system that promotes cartilage repair and regeneration. As chitosan and alginate have different degradation rates, the release rates of tumor growth factor-β (TGF-β) and bone morphogenic protein-7 (BMP-7) are also different. TGF-β is slowly released, while BMP-7 is released more rapidly to simulate physiological dynamics leading to chondrogenesis and cartilage repair [38]. Better control of growth factors and drug release also decreases the need for administering more doses, causing less toxicity [23]. Integrating nanomedicine technology with surgical interventions may provide better clinical outcomes with a more targeted approach, promoting less invasive strategies, decreasing side effects, and reducing material rejection and corrosion.

#### Skin and wound healing

Nanostructures can be used to hasten wound healing and improve scar formation. Nanoparticles can act like a scaffold that attracts and retains cells involved in wound healing. Nanomaterials can mimic cellular characteristics and can thus serve as temporary scaffolds and ideal environments during the formation, organization, and regeneration of new tissues [5]. PEGylated fibrin gel 3D scaffold was designed by Chung et al. [39] to deliver adipocyte stem cells (ADSCs) to the burn wound and found quicker vascularization, mononuclear cell migration, and granuloma tissue generation, resulting in better wound healing. Polyethylene glycol (PEG)-fibrin scaffolds can prevent scar contraction after meshing autografts, decreasing discomfort and improving graft healing and appearance [40]. The expression of genes involved in wound healing can also be altered using nanoparticle delivery systems. The chitosan-coated porous silicon nanoparticles containing Flightless I (Flii) siRNA can silence Flii, which negatively affects wound healing and improves wound healing [41]. Nanomaterials can also be designed to be responsive to the changing biophysico-chemical environment of the wound as the healing progresses. These nanostructures change the configuration to improve the transfer of naturally occurring materials or release appropriate materials incorporated in the nanoparticles to help better tissue healing. These are called smart stimulus-responsive nanostructures [42]. For instance, pH-sensitive DMAEMA/HEMA (dimethylaminoethyl methacrylate 2/hydroxyethyl methacrylate) scaffold swells when the pH decreases during hypoxia. Expansion of this scaffold improves oxygenation, cell infiltration, granulation tissue formation, tissue-regeneration factor, and pro-healing gene expression, angiogenesis, and tissue remodeling [42, 43]. The nanostructures can also be induced to alter configuration using external stimuli. TiO_2_ nanoparticles (NPs) release the intended amounts of reactive oxygen species (ROS) using a specific ultrasound frequency and intensity, as Osumi et al. studied in 2016 [44]. ROS can either enhance cellular response and angiogenesis at physiological levels or help in microbial action at higher levels aiding in wound healing [42].

#### Infection and antimicrobial control

The treatment and prevention of infection remain a huge issue in medicine, as well as in plastic and reconstructive surgery. Antimicrobial resistance continues to increase with various mechanisms such as pathogens escaping from host immunity, pathogen-producing enzymes which inactivate antimicrobial drugs, drug efflux from efflux pumps, and decreased cell permeability of the pathogen [45]. In finding new strategies to overcome this, nanoparticles are being studied for their antimicrobial properties and role as nanocarriers for antimicrobial drug delivery.

Nanomaterials have been shown to reduce biofilm formation, possibly reducing immunogenic and inflammatory responses due to better infection resistance [5]. As an example, a study by Colon et al. [46] showed that the functions of *Staphylococcus epidermidis* were inhibited on nanoscale ZnO and TiO_2_ compared to its micron surface features counterpart. The nanoformulation also decreased bacterial density and colony-forming units [46], suggesting that numerous implant surfaces with nanofeatures may reduce infection [4]. As individual nanoparticles, Tian et al. [47] demonstrated that silver nanoparticles have dose-dependent wound-healing properties such as rapid healing and improved cosmetic appearance, antimicrobial properties, anti-inflammatory properties, and fibrogenic cytokine modulation properties. Nanomaterials and scaffolds composed of nanomaterials in themselves, therefore, exhibit antimicrobial properties.

Nanoparticles can also be used as antimicrobial drug-delivery systems. Therapeutically active controlled-release wound dressings for the treatment of wounds and skin infections from surgery can be created with anesthetics, antimicrobial compounds, anti-inflammatory agents, plasmids, growth factors, proteins, silver particles, and even bacteria and viruses embedded within synthesized long nanofibers through the process of electrospinning [20, 21]. Zhou et al. [48] demonstrated that electrospun wound dressings made from a combination of water-soluble carboxyethyl chitosan and poly-vinyl alcohol showed no toxicity when evaluated in vitro with a mouse fibroblast L929 cell line and even stimulated cell attachment and proliferation. An in vivo study by Dai et al. [49] explored the antimicrobial properties of the engineered chitosan acetate bandage in mice with third-degree burns. Chitosan acetate topical antimicrobial dressing was applied to burns in mice contaminated with *Pseudomonas aeruginosa* and *Proteus mirabilis.* The results showed that the dressing could adhere to the infected wounds for up to 21 days and that the chitosan acetate bandage effectively controlled bacterial growth in the wound and prevented systemic sepsis [49]. The authors also demonstrated that chitosan acetate bandages showed rapid antibacterial action, thus preventing the development of fatal infections when applied to full-thickness excisional wounds in mice infected with *P. aeruginosa*, *P. mirabilis*, and *Staphylococcus aureus*. Wound dressings can also be infused with silver ions (Ag^+^) for their active antimicrobial properties [50]. The major contribution of the antimicrobial property of silver ions is attributed to its interference with thiol groups [50]. As a result, it provokes the generation of ROS [50]. A study by Adhya et al. [51] compared the effectiveness of topical silver sulfadiazine (SSD) and AgNP (silver nanoparticle) hydrogel in burn wound management. Results showed that the healing status of 2° deep-dermal burns at four weeks was more satisfactory for AgNP treatment than SSD (80.6% showed at least 50% healing on AgNP vs. 48.1% on SSD, *p* = 0.001). This makes AgNP an effective and superior alternative to SSD in treating burn wounds, specifically 2° deep-dermal burns [51]. Aside from silver, copper nanoparticles are also studied and are found to have antimicrobial activity [52, 53]. A study by Ramyadevi et al. [52] tested copper nanoparticles on bacteria (*Micrococcus luteus*, *Staphylococcus aureus*, *Escherichia coli*, *Klebsiella pneumoniae*, and *Pseudomonas aeruginosa*) and fungi (*Aspergillus flavus*, *Aspergillus niger*, and *Candida albicans*). Results showed more inhibitory activity in bacteria than in fungi, with a greater zone of inhibition in *E. coli* than in *C. albicans* (26 vs. 23 mm) [52].

Relevant to today’s pandemic, Almanza-Reyes et al. [54] have shown in vivo that silver nanoparticles have an inhibitory effect on SARS-CoV-2 infection in cultured cells. Additionally, in vitro, the study also demonstrated that silver nanoparticles incorporated in mouth and nasal rinses significantly decreased the incidence of SARS-CoV-2 infection in health personnel exposed to patients diagnosed with COVID-19 [54]. As the world and hospitals are slowly opening to the new normal, using these nanoparticles with actions against SARS-CoV-2 may be an excellent addition to pre- and post-PRS care. Overall, nanoparticles show great potential for infection control which can be applied in plastic and reconstructive surgery. However, much is yet to be investigated on the mechanisms, effects, and translatability of nanoparticle-based antimicrobials.

#### Prevention of transplant and graft rejection

Transplant and graft rejection can be prevented by modifying the surface of the graft using nanoparticle coatings that have similar structures or biochemical properties to the host cells or tissues. Nanoparticles can also facilitate tissue regeneration around the graft, thereby protecting it from the host immune response [23]. Implantable immunosuppressant delivery using nanomaterials can also prevent graft rejection. These may provide a localized, targeted, and sustained release of immunosuppressant compounds at the donor-graft site, reducing the dose, frequency of administration, and risk for systemic side effects [22]. Nanoparticles can improve the targeted delivery of immunosuppressive drugs that might be difficult to administer solely for reasons such as, but not limited to, hydrophobicity or hydrophilicity and fast rate of degradation. Tacrolimus, a highly hydrophobic drug, loaded in thermosensitive hydrogel mPEG-PLCL (methoxy-poly(ethylene glycol)-co-poly(lactic acid)-poly(e-caprolactone), was found to be effectively delivered to induce immunosuppression near the skin graft site in rats with minimal effects on systemic immune responses [55]. Nanostructures can also be used to initiate immune tolerance by administering donor antigens to the recipient in a controlled manner before transplantation. Shah et al. [56] showed the effective facilitation of immune tolerance and improved graft protection using poly(lactide-co-glycolide) nanoparticles complexed with ECDI (1-ethyl-3-(3′-dimethylaminopropyl)-carbodiimide) and epitope-containing short peptide. Application of nanoparticles made of and with materials that aid in angiogenesis and immune modulation on the graft site can also increase the survivability of the graft. Bioglass and zinc-doped strontium-substituted bioglass/ceria hybrid nanoparticles treatment on the graft site before flap implantation significantly improved flap survival in rats [57].

#### Three-dimensional printing

3D printing has evolved as a cutting-edge method for maximizing the value of high-resolution tomography pictures and virtual 3D reconstructions [58]. The convergence of different medical fields has led to innovation and advances in the new era of head and neck microsurgical reconstruction. The combination of virtual surgical planning and 3D printing allows hands-on examination and evaluation of surgical techniques, bone transplant placement, aesthetics, and troubleshooting approaches using 3D printed models of a patient-specific craniofacial anatomy or defect [59]. This was proven helpful in congenital cardiothoracic surgery and is now another unique tool for craniofacial surgeons [60]. The integration of virtual surgical planning with 3D printing has increased surgical precision, efficiency, and the ability to deal with more difficult reconstructions, such as with 3D-printed models for pre-bending fixation plates, which demonstrated less operational time and enhanced reconstruction results [59]. Sotsuka et al. [61] pioneered 3D printing to map vasculatures for better comprehension pre-operatively and less complications during surgery in breast reconstructive surgery. Ogunleye et al. (2020) utilized this application to plan the surgery of 58 breast cancer patients, while Mehta et al. (2016) and Jablonka et al. (2019) used this to visualize the abdominal wall containing Deep inferior epigastric (DIEP) vessels and their path concerning neighboring soft tissues [62–64].

Another recent advancement is the rise of 3D bioprinting, which utilizes bio-inks that encapsulate cells, growth factors, and/or nanoparticles to produce 3D scaffolds. Instead of the conventional soaking or seeding of these materials, bio-inks encapsulate them within the matrix to form scaffolds [65]. Gao et al. [66] 3D bioprinted bone tissue with polyethylene glycol dimethacrylate bioink, bone marrow-derived mesenchymal stem cell (BM-MSC), with bioactive glass (BG) nanoparticles, with the latter enhancing the osteogenic potential of the bioink. AgNPs can also be encapsulated in bioinks to confer antibacterial, antioxidant, and osteogenic activities [67]. Overall, using 3D printing in plastic and reconstructive surgery would not only help prepare the surgical team to do the surgery but also help the patient who will undergo surgery.

### Future directions

#### Integration with nanogenerators and implantable electronics

Repetitive and spatiotemporally controlled drug release is an advantageous feature that electrical-stimulation-regulated drug release offers. Nanogenerators and implantable electronics may provide more convenience than current bulky electrical stimulators or implantable ones, which need frequent battery replacement and pose a higher risk of surgical infection.

Ultra-thin films consisting of conformational piezoelectric transducers, a type of nanogenerator, have been shown to map the mechanical characteristics of human skin with excellent fidelity [5, 68]. A piezoelectric nanogenerator (PENG) is a self-powered energy harvester that converts biological energy into electricity, suitable as a power source for wearable or implantable medical devices [69]. It can monitor the spatiotemporal release kinetics of medications given (for example, to the skin), as well as how effectively the wound responds to medical treatments [68]. Furthermore, with the mentioned ultra-thin film, it is hypothesized that the wound healing process may be observed in real-time, allowing for more accurate and regulated pharmacological and surgical therapies. Wirelessly controlled drug-delivery microchips constructed from nanostructures might likewise be implanted in a surgical site and configured to administer specified dosages of medications at predetermined times, reducing the impact of patient noncompliance or missing doses [70]. Moreover, breast implants may one day serve as a tool for cancer monitoring by embedding microelectromechanical system devices inside breast cancer tissue, identifying neoplasms, and informing physicians of recurrences [5, 71]. Graphene, colloidal nanoparticles, and semiconductor nanowires are some of the popular nanomaterials that can be configured to the nanoscale [5].

#### Theranostics

Theranostics is the integration of therapeutics and diagnostics [72]. Nanotheranostics applies nanomedicine methods and uses colloidal nanoparticle agents (10-1000 nm) for advanced theranostics with the goal of diagnosing and treating diseases during the initial stages [73–75]. Advanced nanotheranostic medicine platforms include macromolecular materials/polymers (e.g., drug-polymer conjugates, polymeric nanoparticles, solid-lipid particles, dendrimers, liposomes, micelles, gold nanoparticles, carbon nanomaterials) in which simultaneous integration of the controlled, prolonged, and targeted co-delivery of the diagnostic and therapeutic agents could be designed [73–76]. Applications and advancements in nanotheranostic technologies will potentially lead to improved theranostic benefits and fewer adverse effects [76].

Some of the recent theranostic advances include their application in immuno-oncology and cardiovascular medicine. Nanobody derivatives, with their small size, stability, affinity, and ease of engineering in multiple constructs, are being studied as cancer theranostic agents (e.g., *HER2* targeting nanobodies for breast cancer) [77, 78]. Radiometal-based theranostics or theragnostics have also shown considerable advancements in combining targeted imaging and therapy (e.g., targeting prostate-specific membrane antigen for prostate cancer and somatostatin receptor for neuroendocrine tumors) [78]. In cardiovascular diseases, ultra-small superparamagnetic iron oxide (USPIO) NPs, VCAM-1 internalizing nanoparticles-1 (VINP-1), which, as its name, target the vascular cell adhesion molecule-1(VCAM-1) [79], were able to detect early-stage atherosclerosis at the actual site of plaque formation due to their ability accumulate inside endothelial cells and macrophages [79–83].

Despite the relatively broad literature on theranostics in other fields of medicine, as above, published studies about its application in plastic and reconstructive surgery are limited. One study used the nanotheranostic agent liposomal IR-780 to aid in photothermal/photodynamic therapy (PTT/PDT) in glioblastoma through convection-enhanced delivery (CED). By using this technique, the blood–brain barrier was bypassed, and IR-780 was delivered directly to the glioblastoma tumor, thereby enhancing its anti-cancer property [84]. Furthermore, nanotopography was noted to influence fibroblasts which are vital players in the successful integration of breast implants [85]. In burns and wound care, electrospinning was used to generate nanofibers with anesthetics, antibiotics, and anti-inflammatory agents, potentially treating such patients [20, 21]. It was also found that utilizing silver nanoparticles instead of pure silver sulfadiazine, accelerated wound healing (11 days vs. 26.5 days) in rat models with thermal injury and decreased hypertrophic scarring [47].

## Conclusions

PRS is a dynamic and rapidly progressing field of medicine. However, despite advancements in surgical techniques, several issues remain, such as allograft-related morbidities, the threat of transplant rejection, and immunosuppressive toxicity. Recent studies in nanomedicine and nanoparticle-based delivery systems show promise of addressing these limitations. However, many concepts are still in their preclinical stage investigated with animal models. Further research and clinical trials in humans are necessary for better clinical translation [2]. As of 2016, Anselmo and colleagues reported that over 25 FDA or European Medicines Agency (EMA) approved nanomedicines, and over 45 others are currently undergoing clinical trials. Recently approved preparations included the RNAi therapy Patisiran/ONPATTRO, synergistic two-drug ratio nanoparticle-based delivery system VYXEOS, and radio-enhancing nanoparticle NBTXR3/Hensify. Ongoing clinical trials on nanomedicine and delivery systems are predominantly for cancer, anemia, chronic kidney disease management, and some tools for imaging [86]. Although PRS will likely benefit from nanotechnology applications, especially in oncology and bio-imaging, few direct, novel nanoparticle applications for PRS are being studied and approved (Table 1).

**Table 1:** Summary table of approved nanomedicine/nanoparticle-based delivery system with potential plastics and reconstructive surgery applications

Drug	Company	Application	Active ingredient	Date of approval
Liposome				
Abelcet [87, 88]	Defiante Farmaceutica	Anti-fungal	Amphotericin B	1995
AmBisome [86–88]	Gilead Sciences/NeXstar Pharmaceuticals	Anti-fungal	Amphotericin B	1997
Lipid-based				
Optison [86]	GE Healthcare	Ultrasound contrast agent (e.g., lymph node)	Human serum albumin stabilized perflutren	1997
Definity [86]	Lantheus Medical Imaging	Ultrasound contrast agent (e.g., breast tumors	Perflutren	2001
SonoVue [86]	Bracco Imaging	Ultrasound contrast agent (e.g., breast tumors	Phospholipid-stabilized Sulphur hexafluoride	2001
Nanocrystal				
Vitoss [87, 89]	Orthovita Inc	Bone-grafting material	β-tricalcium phosphate	2003
OsSatura [87]	Isotis Othobiologics Inc	Bone substitute	Hydroxyapatite	2003
Ostim [87, 90]	Osartis GmbH & Co	Bone-grafting material	Calcium hydroxyapatite	2004
NanOss[87, 91]	RTI Surgical	Bone substitute	Hydroxyapatite	2005
EquivaBone [87, 92]	Zimmer Biomet	Bone substitute	Hydroxyapatite	2009

Another critical factor in adopting nanomedicine therapies in clinical practice is incorporating the technology into the current clinical skill set. Platforms should be developed that will allow greater utilization of microsurgical and transplantation skills. This may encourage plastic surgeons to adapt these new technologies into their practice and result in technology translation [2]. By providing avenues and encouraging the use of nanomedicine in PRS, surgeons will be better acquainted with the benefits it offers to the practice. Subsequently, gaining popularity in practice will also propel more research in this field, allowing faster resolution of nanotechnology challenges.

Nanoparticle formulation faces several unique biological, technological, and design-related challenges. In nanoparticle-based delivery systems, challenges in modulating biodistribution and controlling the passage of nanoparticles across biological barriers and into target cells are encountered, especially with the differences in the biological responses of animal models compared to humans [93]. Advanced models such as machine perfusion and organ-in-a-chip systems may have to be used to address the intricacies of the system [2]. To be used clinically, these nanomedicine formulations should also undergo scaled-up synthesis, performance optimization, and performance predictions. Reliable methods on how to do these are important to prevent batch-to-batch variability in the efficacy and safety of the preparation [93]. The unique properties of nanoparticles, advanced models for in vivo assessments, additional steps needed for the preparation of the desired delivery system and foreseeing more significant regulatory hurdles would undoubtedly lead to increased costs of formulation. Therefore, striking a balance between additional functionality and complexity of nanoparticle-based delivery systems is necessary [94]. To our knowledge, cost/benefit studies on these types of formulations have yet to be done. However, ultimately, the justification of the cost of the preparation and use of nanoparticles in PRS would rely on the clinical benefits that will be observed with the first few approved nanomedicines [94]. Nanotechnology, nanomedicine, and nanoparticle-based delivery systems in PRS are still in their infancy. However, their significance in the field is already very apparent. Nanomedicine is a promising and exciting field that will benefit PRS practice and improve patient clinical outcomes.

## Data Availability

The datasets generated during and/or analyzed during the current study are available from the first author on reasonable request.

## Abbreviations

3D: Three-dimensional
ACI: Autologous chondrocyte implantation
ADSC: Adipocyte stem cell
AFM: Atomic force microscope
AgNP: Silver nanoparticle
BG: Bioactive glass
BBB: Blood-brain barrier
BM-MSC: Bone marrow-derived mesenchymal stem cells
BMP-7: Bone morphogenic protein-7
CED: Convection-enhanced delivery
DIEP: Deep inferior epigastric
DMAEMA/HEMA: Dimethylaminoethyl methacrylate 2/hydroxyethyl methacrylate
ECDI: 1-Ethyl-3-(3′-dimethylaminopropyl)-carbodiimide
ECM: Extracellular matrix
EMA: European Medicines Agency
EpCAM: Epithelial Cellular Adhesion Molecule
EV: Extracellular vesicle
FDA: Food and Drug Authority
FGF-2: Fibroblast growth factor-2
Flii: Flightless I
IND: Investigational new drug
MACI: Matrix-induced autologous chondrocyte implantation
mPEG-PLCL: Methoxy-poly(ethylene glycol)-co-poly(lactic acid)-poly(e-caprolactone)
NDA: New drug application
NP: Nanoparticle
nPTFE: Nanostructured polytetrafluoroethylene
PEG: Polyethylene glycol
PENG: Piezoelectric nanogenerator
PET: Positron emission tomography
PLK1: Polo-like kinase 1
POSS-PCU: Polyhedral oligomeric silsesquioxane poly(carbonate-urea) urethane
PRS: Plastic and reconstructive surgery
PTT/PDT: Photothermal/photodynamic therapy
ROS: Reactive oxygen species
SLN: Sentinel lymph nodes
SSD: Silver sulfadiazine
STM: Scanning tunneling microscope
TGF-β: Tumor growth factor-β
USPIO: Ultra-small superparamagnetic iron oxide
VCAM-1: Vascular cell adhesion molecule 1
VINP: VCAM-1 internalizing nanoparticles
